# Increased extracellular vesicle miRNA-466 family in the bronchoalveolar lavage fluid as a precipitating factor of ARDS

**DOI:** 10.1186/s12890-019-0876-9

**Published:** 2019-06-20

**Authors:** Sotaro Shikano, Yasuhiro Gon, Shuichiro Maruoka, Tetsuo Shimizu, Yutaka Kozu, Yuko Iida, Mari Hikichi, Mai Takahashi, Shinichi Okamoto, Kota Tsuya, Asami Fukuda, Kenji Mizumura, Shu Hashimoto

**Affiliations:** Division of Respiratory Medicine, Department of Internal Medicine, Nihon University School of Medicine, Tokyo, Japan

**Keywords:** ARDS, Extracellular vesicles, miRNA, NLRP3 inflammasome

## Abstract

**Background:**

Acute respiratory distress syndrome (ARDS) is a life-threatening disease; however, its treatment has not yet been fully established. The progression of ARDS is considered to be mediated by altered intercellular communication between immune and structural cells in the lung. One of several factors involved in intercellular communication is the extracellular vesicle (EV). They act as carriers of functional content such as RNA molecules, proteins, and lipids and deliver cargo from donor to recipient cells. EVs have been reported to regulate the nucleotide-binding oligomerization like receptor 3 (NLRP3) inflammasome. This has been identified as the cellular machinery responsible for activating inflammatory processes, a key component responsible for the pathogenesis of ARDS.

**Methods:**

Here, we provide comprehensive genetic analysis of microRNAs (miRNAs) in EVs, demonstrating increased expression of the miRNA-466 family in the bronchoalveolar lavage fluid of a mouse ARDS model.

**Results:**

Transfection of bone marrow-derived macrophages (BMDMs) with miRNA-466 g and 466 m-5p resulted in increased interleukin-1 beta (IL-1β) release after LPS and ATP treatment, which is an established in vitro model of NLRP3 inflammasome activation. Moreover, LPS-induced pro-IL-1β expression was accelerated by miRNA-466 g and 466 m-5p in BMDMs.

**Conclusions:**

These findings imply that miRNA-466 family molecules are secreted via EVs into the airways in an ARDS model, and this exacerbates inflammation through the NLRP3 inflammasome. Our results suggest that the NLRP3 inflammasome pathway, regulated by extracellular vesicle miRNA, could act as a therapeutic target for ARDS.

## Background

Acute respiratory distress syndrome (ARDS) is an acute, diffuse, inflammatory form of lung injury [1]. Primarily, direct injuries include severe pneumonia and aspiration pneumonia, whereas the predominant indirect injuries are sepsis and shock. Sepsis is defined as systemic inflammatory response syndrome (SIRS) accompanied by infection [2]. SIRS induces the overproduction of inflammatory cytokines and causes multiple organ dysfunction syndrome (MODS); ARDS is considered a component of MODS [3]. A cohort study analyzing patients who visited the ICU reported mortality rates to be 34, 40, and 46% for mild, moderate, and severe ARDS, respectively [4]. Although mechanical ventilation with a lower tidal volume results in decreased mortality and increases the number of days without ventilator use in patients with ARDS [5], several other translational clinical ARDS studies failed to show a reduction in mortality rates [6]. These reports suggest that ARDS is a life-threatening disease. Furthermore, no treatment has proven to be sufficiently effective in preventing ARDS.

The pathophysiology associated with ARDS, involves 1) activated macrophages overproducing inflammatory cytokines such as interleukin-1 beta (IL-1β) and interleukin-8 (IL-8), which activate neutrophils that have difficulty in passing through microvessels in the lung; 2) vascular endothelia in the lung overproduce adhesion molecules, and these phenomena result in neutrophil accumulation in the lung vessels; and 3) under the influence of chemoattractants released by macrophages, neutrophils migrate into the lung interstitium or pulmonary alveoli where they produce neutrophil elastase and reactive oxygen species, resulting in alveolar epithelial cell injuries [7]. Therefore, a neutrophil elastase inhibitor is a key therapeutic target for ARDS; however, it only has partial effects on this disease. Hence, the development of new therapies for the treatment of ARDS is urgently needed.

Extracellular vesicles (EVs) are lipid bilayer-covered vesicles with a diameter of 40–100 nm that are found in body fluids [8]. EVs mainly contain mRNAs, microRNAs (miRNAs), and proteins [9]. miRNAs are small RNA molecules comprised of 21–25 nucleotides that exist in many organisms [10]. They participate in homeostasis by regulating protein expression via the degradation of mRNA or suppression of translation after binding to target mRNA [11]. Lotvall et al. reported that extracellular vesicle miRNAs from mouse mast cells can be transferred to human mast cells resulting in the production of mouse protein [12]. Given that the progression of ARDS is mediated by altered intercellular communication in the lung, between immune and structural cells [13], EVs and their contents are promising candidates for targeted ARDS therapy.

The NLRP3 inflammasome is a protein complex that activates caspase-1, a protease, and induces IL-1β secretion in macrophages [14]. The inflammasome is composed of two pathways, namely priming and triggering. In the priming pathway, toll like receptor 4 (TLR4) in macrophages recognizes pathogen-associated molecular pattern (PAMP) or damage-associated molecular pattern (DAMP) molecules resulting in the production of pro-IL-1β [15]. TLR4-mediated production of pro-IL-1β is regulated by the zinc finger protein A20 in the airway [16]. In triggering this pathway, the P2X7 receptor in macrophages recognizes adenosine triphosphate (ATP), a danger signal released extracellularly after cell injury, activates caspase-1 through the NLRP3 inflammasome, and produces IL-1β from pro-IL-1β [14]. Dolinay et al. reported that serum IL-1β levels can be increased by mechanical ventilation in patients who contracted sepsis accompanied by ARDS [17]. However, the molecular mechanism underlying the regulation of the inflammasome in ARDS is not fully understood. As EVs have been reported to regulate the NLRP3 inflammasome [18], we hypothesized that EV miRNA may regulate the NLRP3 inflammasome pathway and contribute to the pathophysiology of ARDS.

In this study, we show that expression of miRNA-466 g and miRNA-466 m-5p is increased in EVs that are secreted into the airways of mice after intratracheal administration of LPS. Transfection of bone marrow-derived macrophages (BMDMs) with miRNA-466 g and 466 m-5p resulted in increased IL-1β release after treatment with LPS and ATP, which is an established in vitro model of NLRP3 inflammasome activation. Furthermore, LPS-induced pro-IL-1β expression was accelerated by miRNA-466 g and 466 m-5p in BMDMs. Our study suggested that EV miRNA-466, which is secreted into the airways, can exacerbate inflammation in a mouse ARDS model via the NLRP3 inflammasome, and thus this pathway might represent a therapeutic target for ARDS.

## Methods

### Reagents

The following antibodies were used: goat anti-mouse IL-1β (clone AF-401-NA; R&D systems, Minneapolis, MN, USA); rabbit anti-mouse β-actin (#4967; Cell Signaling Technology, Tokyo, Japan); donkey HRP-linked anti-goat IgG (sc2020; Santa Cruz Biotechnology, Dallas, TX, USA); goat HRP-linked anti-rabbit IgG (#7074; Cell Signaling Technology, Tokyo, Japan). LPS derived from O55:B5 *Escherichia coli* (L-2880), ATP (A-2383), and the NLRP3 inflammasome inhibitor CP-456773 were purchased from SIGMA-Aldrich (St. Louis, MO, USA).

### Animals and EV extraction

All animal care and experimental procedures used in the present study were approved by the Animal Care and Use Committee at Nihon University School of Medicine. Mice were housed in a temperature-controlled conventional room and supplied with laboratory chow and water ad libitum. We purchased C57BL/6 J male mice from Charles River (Yokohama, Japan). We prepared two groups of 8-week-old mice; after anesthesia with 2% isoflurane, one group was treated with intratracheal administration of 50 μL PBS, whereas the other group received 50 μL of a 1 μg/μL LPS solution. Twenty-four hours later, mice were euthanized with CO_2_ and received 1 mL of PBS intratracheally. BALF samples were obtained from both groups. All procedures were performed in such a way as to minimize animal suffering. BALF was centrifuged at 4 °C at 500×*g* for 10 min, and the supernatant was harvested. We extracted RNA from EVs using the Sera MirTM Exosome RNA Amplification Kit (System Biosciences, Palo Alto, CA, USA). We observed the morphology of EVs under a scanning electron microscope and counted the number of EVs using qNano (Meiwafosis, Tokyo, Japan). qNano measures particles using the Tunable Resistive Pulse Sensing (TRPS) principle, in which nanoparticles suspended in electrolytes are detected on a particle-by-particle basis as they pass through a nanopore. We measured the amount and size of RNA using the Agilent RNA6000 pico kit (Agilent Technologies, Santa Clara, CA, USA).

### Comprehensive genetic analysis

EVs extracted from the BALF of 10 mice were pooled to comprise one sample to obtain sufficient amounts of RNA for comprehensive genetic analysis. RNA was labeled by Affymetrix Flash TaqTM Biotin HSR RNA Labeling Kits for Gene Chip® and applied to miRNA3.0 Array (Affymetrix, Santa Clara, CA, USA). We analyzed converted fluorescence intensity using the GeneChip Fluidics Station 450 and GeneChip scanner 3000 (Affymetrix, Santa Clara, CA, USA). The data were statistically analyzed with Partek Genomics Suite (Partek Inc., St. Louis, MO, USA). The data were subjected to principle component analysis using Partek Genomics Suite software for microarray quality control.

### Real-time PCR analysis

We synthesized complementary DNA (cDNA) from RNA that was extracted from EVs of mouse BALF using the TaqMan MicroRNA Reverse Transcription Kit (Applied Biosystems, Foster City, CA, USA). Real-time quantitative PCR was performed with an Applied Biosystems 7300 standard real-time PCR system (Applied Biosystems). We used miRNA-466 g (Assay ID 241015_mat, Applied Biosystems), miRNA-466 m-5p (Assay ID 465329_mat, Applied Biosystems), and U6 (Assay ID 19173, Applied Biosystems), an endogenous standard, as TaqMan primers. The Ct values were determined by the initial threshold, and the amount of RNA was evaluated by the comparative ΔΔCT method.

### Cell culture

We extracted bone marrow from the femur and tibia of C57BL/6 J mice. Cells were cultured in L929 cell conditioned medium until differentiation into BMDMs. The concentration of the supernatant of L929 cell conditioned medium was 30% for day 1 to 2 after the bone marrow was extracted, and 25% for day 3 to 7. We cultured BMDMs in Dulbecco’s modified Eagle’s medium (DMEM) - low glucose (SIGMA-Aldrich), containing 10% fetal bovine serum (FBS) (SAFC Biosciences, Lenexa, KS, USA), 1% penicillin-streptomycin (Nacalai Tesque, Kyoto, Japan), and L929 cell conditioned medium at 100% humidity and 37 °C.

### Transfection

We transfected 50 nM of miRNAs into BMDMs cultured for 7 days on 24-well plates using Lipofectamine RNAiMAX (Life Technologies, Yokohama, Japan). We used miRNA mimic Negative Control #2 (Bioneer, Daedeok-gu, Korea), MMU-MIR-466 g mimic (Bioneer, Daedeok-gu, Korea), and MMU-MIR-466 m-5p mimic (Bioneer, Daedeok-gu, Korea). After transfection, we cultured BMDMs with DMEM - low glucose (SIGMA-Aldrich), containing of 10% FBS (SAFC Biosciences) and 20% L929 cell conditioned medium at 100% humidity and 37 °C.

### ELISA

We collected the BMDM supernatant, without centrifugation or dilution, and measured mouse IL-1β using an ELISA kit purchased from Affymetrix (Santa Clara, CA, USA) in accordance with the manufacturer’s instructions.

### Western blotting

We extracted proteins from BMDMs using radioimmunoprecipitation assay buffer. Lysates were boiled for 10 min in sample-loading buffer (XT sample buffer 4x and XT reducing agent 20x mix; BIO RAD, Hercules, CA, USA). Proteins were separated by electrophoresis using criterion XT Bis-Tris precast gels (4–12%) (BIO RAD, Hercules, CA, USA) and transferred to PVDF membranes (immobilon; Millipore, Burlington, MA, USA) by electroblotting. Samples containing 20 μg of protein were used for electrophoresis. The bands were detected using Amersham ECL Prime Western Blotting Detection Reagent (GE Healthcare, Tokyo, Japan) and a luminescence image analyzer LAS-4000IR (Fuji Film, Tokyo, Japan).

### Statistical analysis

Data were analyzed with Graph pad prism 6 (GraphPad Prism Software, La Jolla, CA, USA) and provided as mean ± standard deviation (SD) or mean ± standard error (SE). Significant differences between groups were indicated at *P* < 0.05 after performing a student’s t - test or ANOVA test with Tukey’s multiple comparison test.

## Results

### Intratracheal administration of LPS increases EV RNA in mouse BALF

**Fig. 1:**
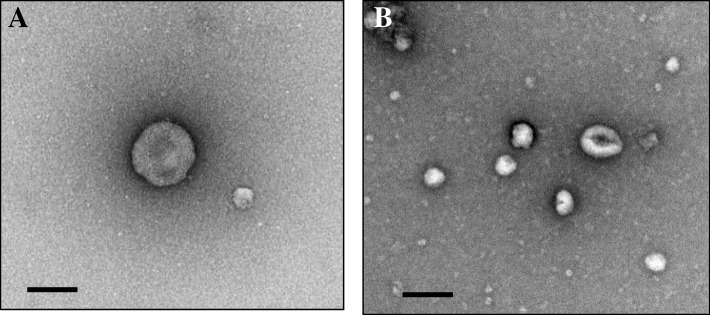
Scanning electron microscopy image of BALF EVs. We observed BALF EVs extracted from mice who received intratracheal instillation of PBS (**a**) or LPS (**b**) by scanning electron microscopy (× 10,000). The scale bars correspond to 100 nm in the PBS and LPS groups

**Fig. 2:**
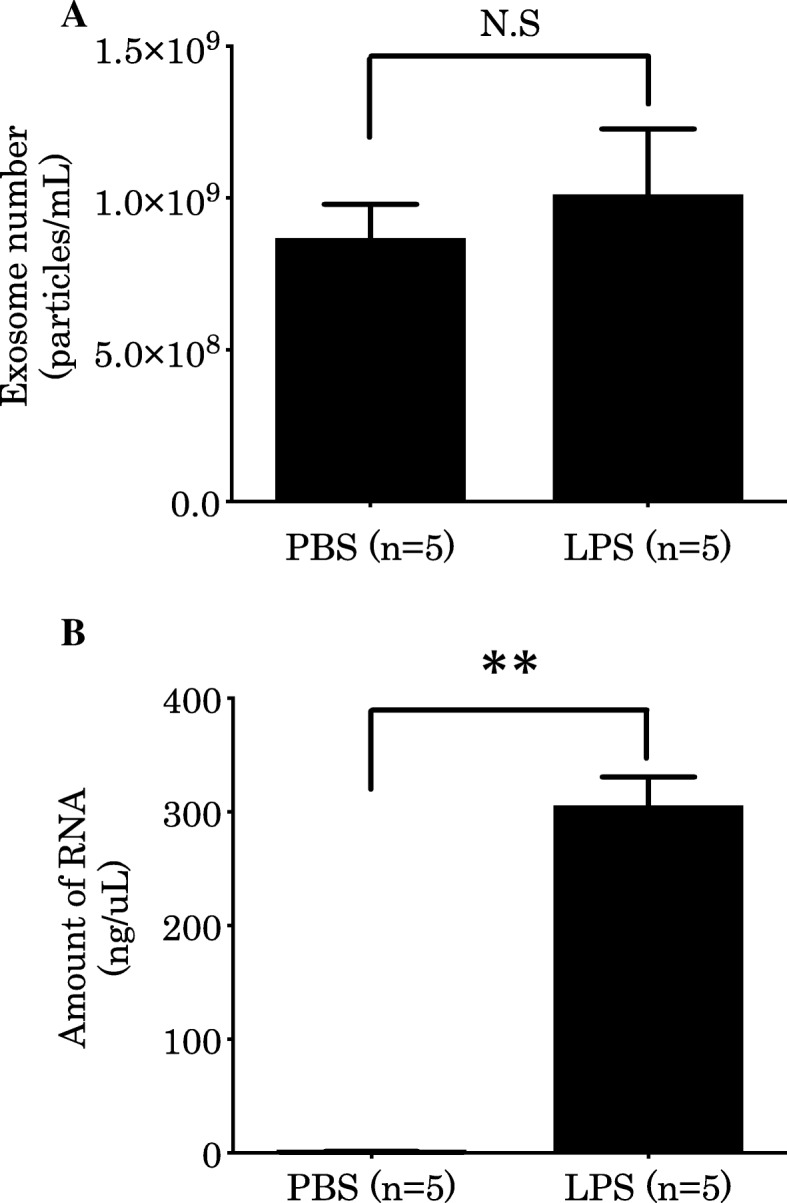
Number of BALF EVs and amount of RNA after LPS stimulation. **a** The number of BALF EVs after LPS stimulation. We counted the number of BALF EVs using a nanoparticle counter (mean ± SD, *n* = 5, N.S = not significant). **b** The amount of RNA after LPS stimulation. We extracted total RNA from BALF EVs and measured RNA amounts (mean ± SD, *n* = 5, **P < 0.01)

First, we assessed changes in the size and number of EV in the BALF from C57BL/6 J mice after intratracheal administration of LPS. Scanning electron microscopy revealed the presence of 100–200-nm particles covered with a lipid bilayer membrane, in both the PBS and LPS group, based on acetic uranyl staining (Fig. 1). Enumeration of nanoparticles revealed that the average number of EV in the PBS group was 0.87 × 10^9^ particles/mL and that in the LPS group was 1.0 × 10^9^ particles/mL (Fig. 2a). However, no significant difference was detected between these groups.

**Fig. 3:**
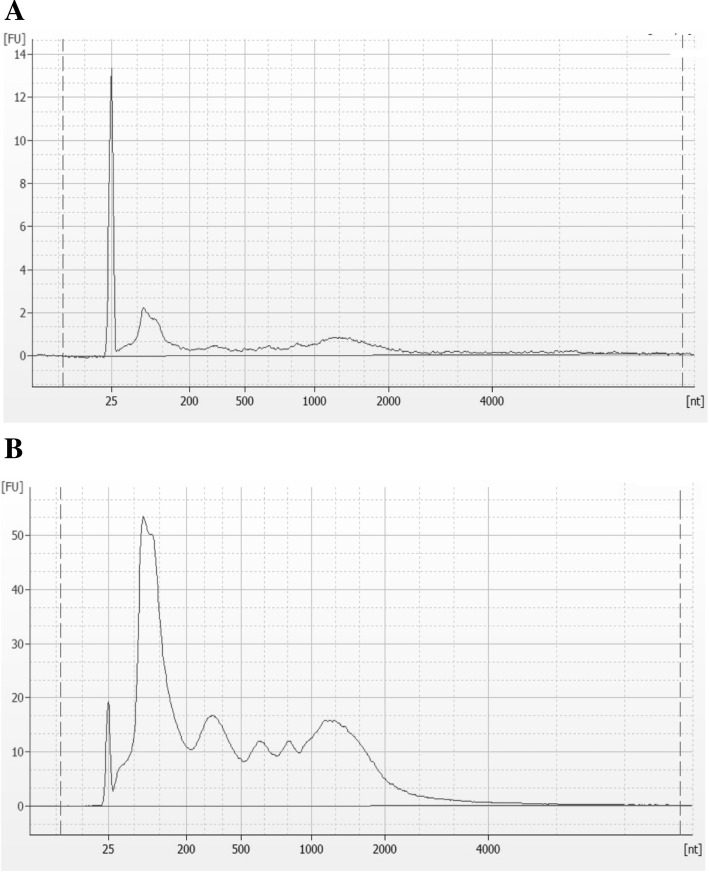
Size of EV RNA. The size of EV RNA from PBS (**a**) or LPS (**b**) -stimulated mice was measured. Nt = nucleotide. FU = fluorescence units

Next, we quantitated the total RNA amount harvested from EVs using the bioanalyzer. The average amount of total RNA in the PBS group was 1.08 ng/μL, whereas that in the LPS group was 305 ng/μL, indicating that LPS stimulation induced a 280-fold increase of total EV RNA (Fig. 2b). The sizes of EV RNAs from the PBS and LPS groups showed that RNA species were 50–100 base pairs in both groups, which is consistent with the size of miRNA (Fig. 3a and b). These results suggested that there were no changes in the number and morphology of EVs, but that the amount of EV RNA, especially miRNA, was notably increased after LPS stimulation.

### Intratracheal administration of LPS increases expression of EV miRNA-466 family in mouse BALF

**Fig. 4:**
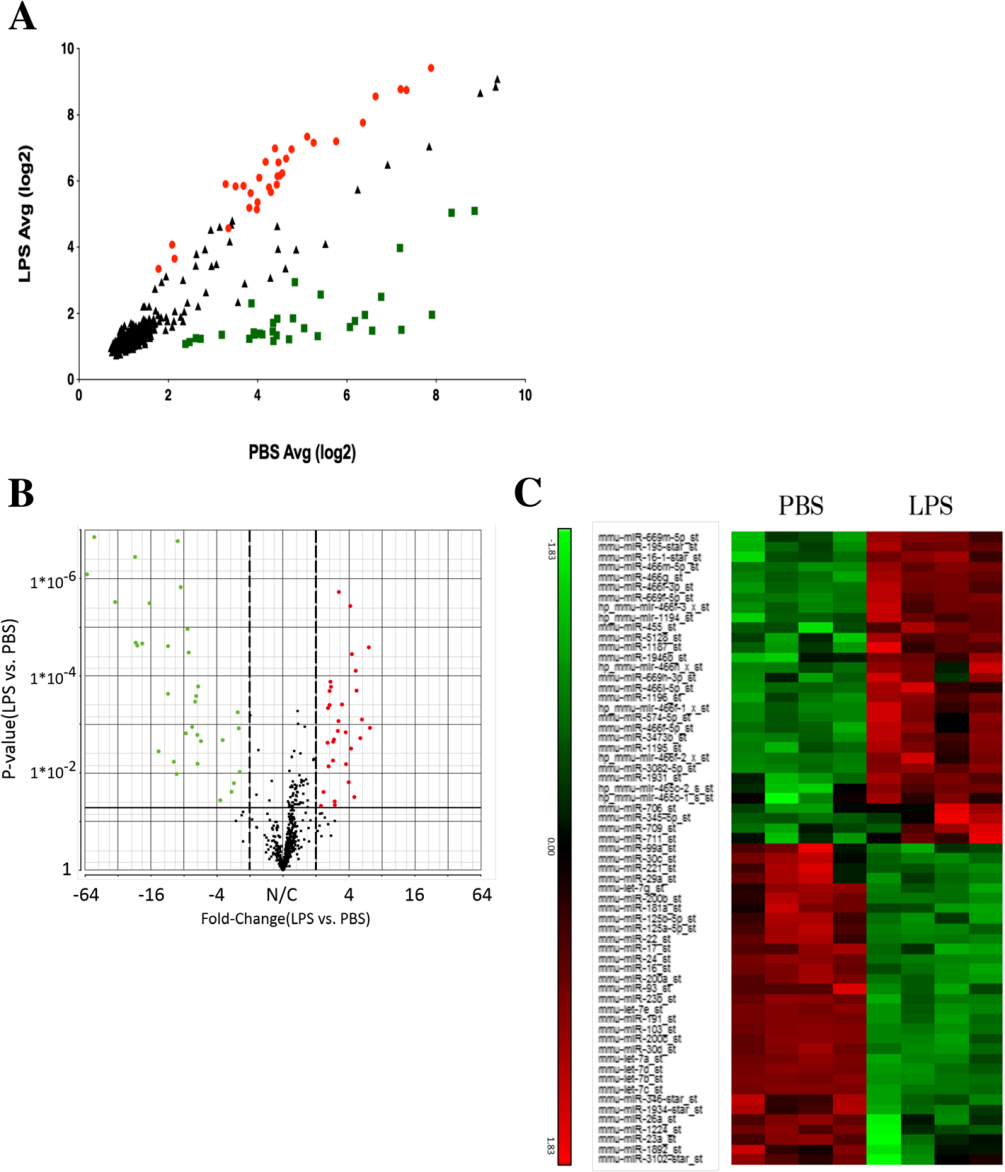
Comprehensive genetic analysis of miRNAs in BALF EVs based on microRNA microarray. Scatter plot (**a**) and Volcano plot (**b**) of gene expression in BALF from LPS and PBS group mice. Red plots show *P* < 0.05 and fold-change > 2. Green plots show P < 0.05 and fold-change < − 2. **a** The value of x and y axes were normalized by log2 scale. **b** Bold line shows *P* = 0.05. Dotted lines show fold-change = − 2 or 2. **c** Heat map of differentially expressed miRNAs. Red and green regions indicate upregulated and downregulated genes, respectively. Each group comprised four mice

**Fig. 5:**
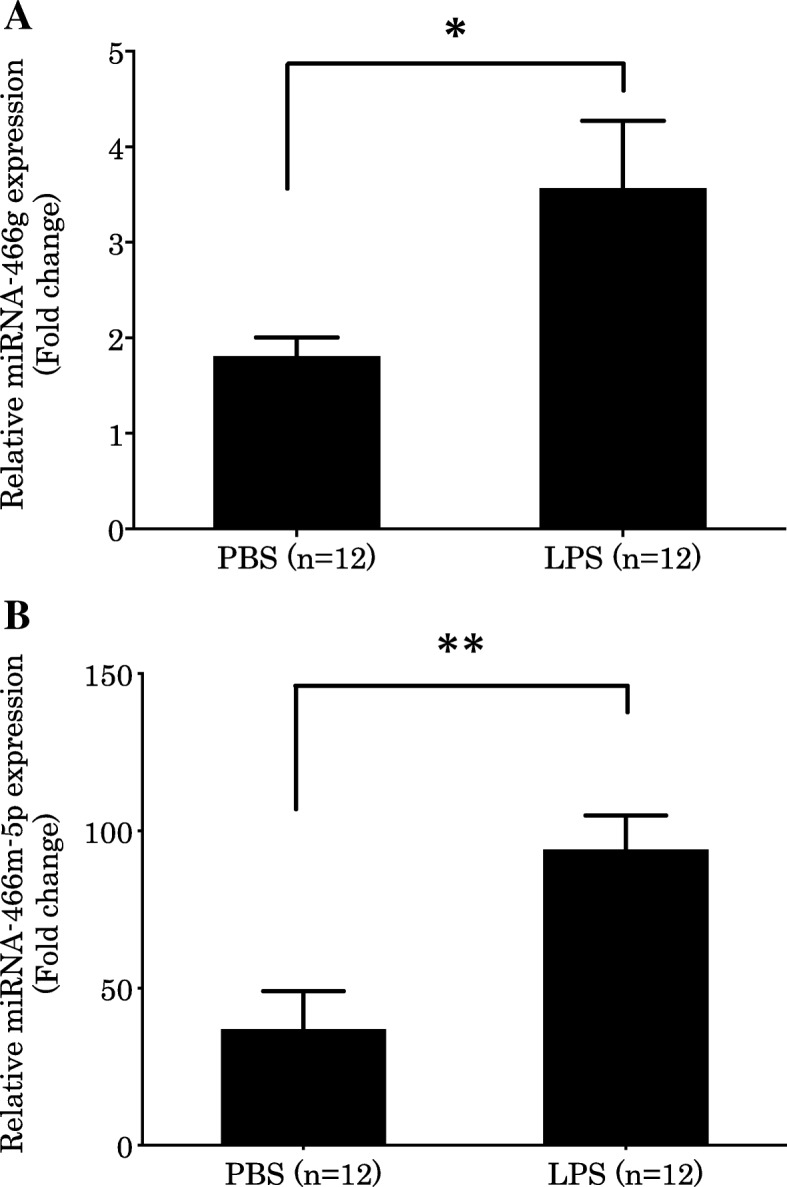
Validation of miRNA-466 g and miRNA-466 m-5p expression in BALF by real-time PCR analysis. BALF was collected from mice who received intratracheal instillation of PBS or LPS. We validated (**a**) miRNA-466 g and (**b**) miRNA-466 m-5p gene expression in mouse BALF by real-time PCR analysis. U6, a small nuclear RNA, was used as an internal standard (mean ± SD, *n* = 12, **P < 0.01, *P < 0.05)

In these experiments, we extracted EV RNA from BALF using EV isolation kit and subjected these samples to comprehensive genetic analysis comprising a miRNA microarray. We used specific fold-change (fold-change> 2) and *P* values (*P* < 0.05) to filter differentially expressed transcripts. The expression of 31 of 597 miRNAs (red dots) was significantly increased and the expression of 32 of 597 miRNAs (green dots) was significantly decreased in the LPS group compared to those in the PBS group (Fig. 4a and b). Heat maps of the differentially expressed miRNAs were generated (Fig. 4c). The top 10 genes of the significantly increased miRNAs contained several genes of the miRNA-466 family (Table 1). Because miR-669 m-5p and miRNA-466 m-5p have the same sequence, we focused on the role of EV miRNA-466 family molecules, especially the top two miRNA-466, miRNA-466 g and miRNA-466 m-5p, in a mouse ARDS model. We validated the expression of miRNA-466 g and miRNA-466 m-5p in BALF of LPS-treated mouse by real-time PCR analysis. We confirmed that the expression of these genes was significantly increased after LPS stimulation (Fig. 5a and b).

**Table 1:** The top 10 miRNAs induced by LPS

miRNA	Fold Change	*p*-value
Mmu-miR-16-1-star	6.17	0.00119
Mmu-miR-466 g	6.04	2.55E-05
Mmu-miR-669 m-5p	5.27	0.000802
Mmu-miR-466f-5p	5.03	0.00193
Mmu-miR-195-star	4.70	0.000202
Mmu-miR-466f-3p	4.60	8.00E-05
Mmu-miR-669f-5p	4.27	3.63E-05
Mmu-miR-1187	4.17	0.00316
Mmu-miR-466 m-5p	4.12	3.62E-06
Mmu-miR-455	3.74	0.00147

A comprehensive expression analysis of miRNAs in EVs from BALF of PBS (*n* = 40) or LPS (*n* = 4) treated mice was performed. The top 10 miRNAs upregulated by LPS are shown. The fold-change represents the ratio of LPS-treated to untreated groups

### The inflammasome is activated by miRNA-466 g and miRNA-466 m-5p through increases in pro-IL-1β during the priming phase

**Fig. 6:**
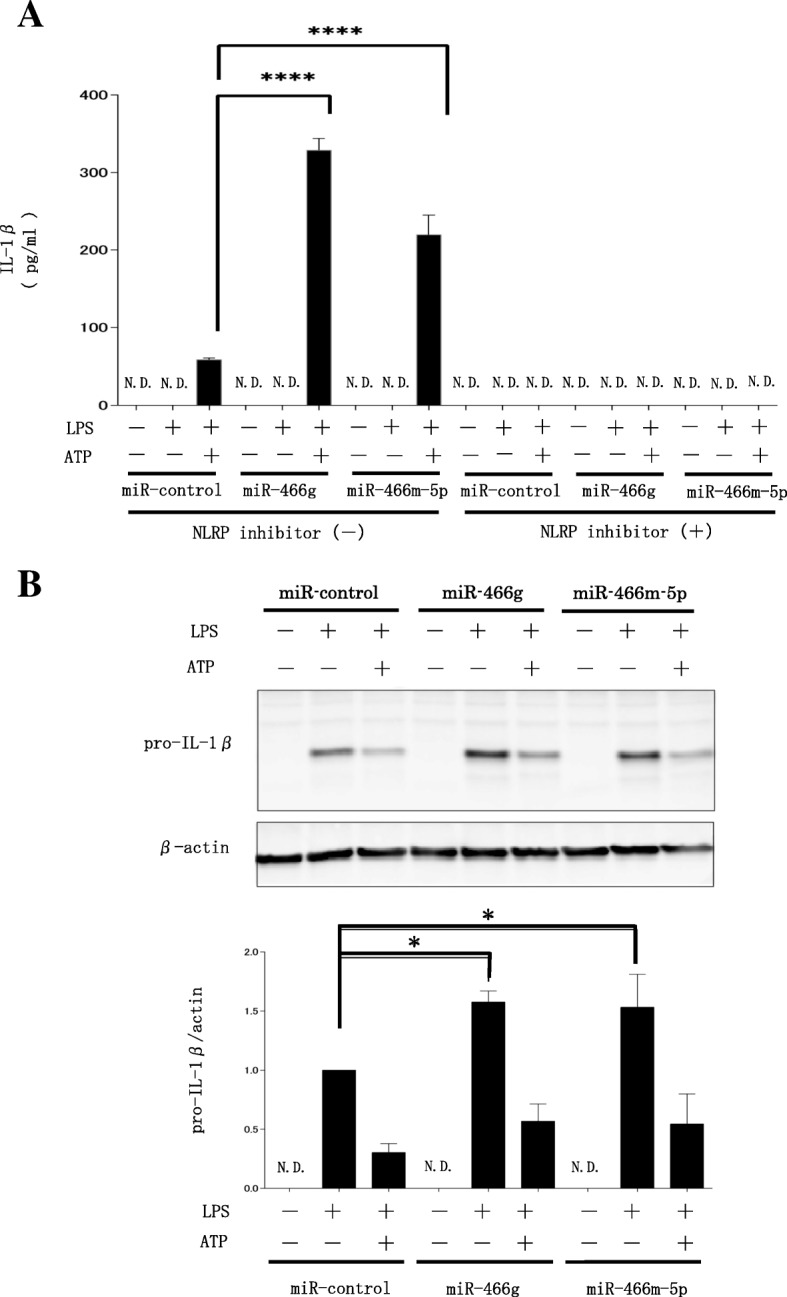
Effects of miRNA-466 g and miRNA-466 m-5p on the production of IL-1β by BMDMs. BMDMs were transfected with miRNA-466 g and miRNA-466 m-5p. Two days after transfection, cells were treated with 10 ng/mL LPS. Five hours later, 1 μM of the NLRP3 inhibitor CP-456773 was added to the culture medium, and 1 h later these cells were stimulated with 5 mM ATP. **a** IL-1β concentration in medium as measured by ELISA (mean ± SD, *n* = 3, ****P < 0.001, N. D. = not detected). **b** Intracellular pro-IL-1β (31 kDa) was detected using western blotting. Pro-IL-1β expression was assessed with densitometry of immunoblots. Signal intensities were normalized to β-actin (mean ± SD, n = 3, *P < 0.05, N. D. = not detected)

To examine the role of miRNA-466 family molecules in inflammasome activation, we transfected BMDMs with miRNA-466 g or miRNA-466 m-5p. Two days after transfection, the cells were stimulated by LPS and ATP with or without the NLRP3 inhibitor CP-456773. ATP-mediated activation of caspase-1 in LPS-primed macrophages is an established in vitro model of NLRP3 inflammasome activation [19, 20]. After treatment with LPS and ATP, the concentration of extracellular IL-1β increased in the supernatant of BMDMs transfected with miRNA-466 g or miRNA-466 m-5p compared to that in cells transfected with a miRNA-negative control (Fig. 6a). Furthermore, the treatment with CP-456773 completely suppressed the LPS/ATP-induced IL-1β production in our experiments. These results indicate that miRNA-466 g and miRNA-466 m-5p are precipitating factors for the inflammasome. The inflammasome is composed of two pathways, namely priming and triggering; thus, we evaluated the role of miRNA-466 g and miRNA-466 m-5p in these pathways. We assessed the effect of miRNA-466 g and miRNA-466 m-5p on LPS-induced pro-IL-1β expression in BMDMs to evaluate the priming pathway. Western blot analysis revealed that LPS-induced intracellular pro-IL-1β expression was significantly accelerated by miRNA-466 g and 466 m-5p transfection compared with miR-control (Fig. 6b). On the other hand, pro-IL-1β expression decreased after LPS-ATP stimulation; and there was no significant change among three groups, suggesting ATP triggering produces IL-1β from pro-IL-1β intracellularly and release it extracellularly. These findings indicate that miRNA-466 g and 466 m-5p might upregulate the priming pathway of inflammasome in BMDM and have a critical role in inflammasome activation.

## Discussion

It is generally accepted that ARDS is caused by inflammation, which is mediated by neutrophil activation [7]. However, the effects of neutrophil elastase inhibition are not sufficient to treat ARDS [21, 22]. Hence, additional studies are required to identify other inflammatory pathways involved in the onset of this disease. As such, we focused on the relationship between ARDS and inflammation via the NLRP3 inflammasome, and analyzed EVs that have been reported to regulate this process [23, 24]. It is known that EVs mediate cell–cell communications in lung inflammation and injury [25]. EVs are highly enriched in cytokines, chemokines, protein, and miRNAs, and target cells could receive the necessary concentration of these inflammation-related molecules via EVs. Although it has been reported that EVs in BALF of mice are rich in miRNA and that miRNA-enriched epithelial EVs trigger macrophage-mediated pro-inflammatory effects [26], the underlying mechanisms remain to be elucidated. We found that expression of the miRNA-466 family molecules were enhanced in EVs isolated from the BALF of LPS-stimulated mice (Fig. 4 and Table 1). Furthermore, miRNA-466 g and miRNA-466 m-5p, which expression was validated by real-time PCR (Fig. 5), were transfected into BMDMs, followed by LPS and ATP stimulation, which induced an increase in IL-1β (Fig. 6a). LPS-induced pro-IL-1β expression was accelerated by miRNA-466 g and 466 m-5p (Fig. 6b). These findings indicate that EV miRNA-466 family molecules are secreted into the airways during ARDS, and that these molecules exacerbate inflammation via the NLRP3 inflammasome. In addition to important cellular functions, secreted EV miRNAs could serve as therapeutic targets for ARDS by inhibiting or eliminating EVs.

In this study, we compared the number of EVs and the amount of EV RNA in the BALF from LPS- or PBS-stimulated mice (Fig. 2). Although there was no significant difference in the number of EVs after LPS administration (Fig. 2a), the amount of EV RNA was significantly increased (Fig. 2b). Furthermore, we showed that EV RNAs from the BALF are small molecules (50–200 bp), suggesting the existence of miRNA (Fig. 3). Given that miRNA dramatically increased after LPS stimulation, this stimulus could result in the sorting of miRNAs into EVs, rather than EV biogenesis and release; which may be necessary to control the biological response to pathogenic microorganisms. However, little is known about whether and how cargo is sorted into vesicles, and thus further investigation is needed to explore the effect of LPS on the sorting of cargo, and especially miRNA, into EVs in the lung.

Next, we analyzed the expression of EV miRNAs in mouse BALF (Fig. 4). We identified 23 miRNA genes that were upregulated after LPS stimulation. Interestingly, the top 10 miRNAs belonged to the miRNA-466 family (Table 1). Several studies have been performed with regards to miRNA-466 expression and its regulatory roles [27, 28]. miRNA-466 was shown to inhibit tumor growth and bone metastasis in prostate cancer [27]. Furthermore, miRNA-466 g was found to regulate sodium and glucocorticoid regulated kinase 1 mRNA expression and epithelial sodium channel activity in the cortical collecting duct [29]. However, the role of miRNA-466 in LPS-induced ARDS was previously unknown. LPS is a known activator of the NLRP3 inflammasome. Moreover, it has been reported that the NLRP3 inflammasome is related to ARDS [23]. Therefore, in this study, we focused on the effect of the miRNA-466 family on the NLRP3 inflammasome. miRNA-466 g and 466 m-5p, transfected into BMDMs, facilitated the secretion of IL-1β after stimulation with LPS and ATP (Fig. 6a). LPS-induced intracellular pro-IL-1β production was accelerated by the transfection with miRNA-466 g or 466 m-5p (Fig. 6b). Our data indicated that miRNA-466 g and 466 m-5p act as precipitating factors for the inflammasome by regulating the priming pathway.

However, our study had several limitations. First, we did not establish whether EV miRNA-466 family molecules aggravate lung injury of mouse ARDS model in this study, whereas our data is consistent with previous study demonstrating that GW4869, an inhibitor of EV release, decreased serum levels of IL-1β in a sepsis-induced inflammation [30]. We also did not investigate the function of all miRNAs that were upregulated in EVs from the mouse ARDS model. As other candidates, in addition to the miRNA-466 family, might have an important role in lung injury, further investigation is needed to evaluate their significance.

## Conclusion

Our study showed that LPS stimulation promoted the expression of miRNA-466 g and miRNA-466 m-5p in EVs that were secreted into the airways. miRNA-466 g and 466 m-5p affected the priming pathway in the macrophage NLRP3 inflammasome. Therefore, IL-1β overproduction by miRNA-466 g and 466 m-5p might be associated with the onset of ARDS. It is plausible that the regulation of exosomes in the BALF or exosomal miRNAs might be a novel therapeutic target for ARDS treatment.

## Data Availability

The datasets used and/or analyzed during the current study are available from the corresponding author on reasonable request.

## Abbreviations

ARDS: Acute respiratory distress syndrome
ATP: Adenosine triphosphate
BMDMs: Bone marrow-derived macrophages
cDNA: Complementary DNA
DAMP: Damage-associated molecular pattern
EV: Extracellular vesicle
IL-1β: Interleukin-1 beta
IL-8: Interleukin-8
miRNAs: microRNAs
MODS: Multiple organ dysfunction syndrome
NLRP3: Nucleotide-binding oligomerization like receptor 3
PAMP: Pathogen-associated molecular pattern
SD: Standard deviation
SIRS: Systemic inflammatory response syndrome
TLR4: Toll like receptor 4
